# The 96-week outcomes and pharmacokinetics of long-acting cabotegravir plus rilpivirine in South Africans

**DOI:** 10.4102/sajhivmed.v26i1.1709

**Published:** 2025-07-22

**Authors:** Rosie Mngqibisa, Yashna Singh, Catherine Orrell, Johan Lombaard, Sandy Griffith, Conn Harrington, Ronald D’Amico, William Spreen, Marty St Clair, Christine Latham, Louise Garside, Rodica Van Solingen-Ristea, Veerle Van Eygen, Fafa Addo Boateng, Herta Crauwels, Prosperity Eneh, Ingrid Eshun-Wilsonova

**Affiliations:** 1 Ward D1, Enhancing Care Foundation, Wentworth Hospital, Durban, South Africa; 2 Desmond Tutu HIV Centre, Institute of Infectious Diseases and Molecular Medicine, Department of Medicine, University of Cape Town, Cape Town, South Africa; 3 Josha Research, Bloemfontein, South Africa; 4 ViiV Healthcare, Durham, North Carolina, United States; 5 Phastar, London, United Kingdom; 6 The Janssen Pharmaceutical Companies of Johnson and Johnson, Beerse, Belgium; 7 Johnson & Johnson Middle East, FZ-LLC, Accra, Ghana; 8 Janssen Scientific Affairs, LLC, Titusville, New Jersey, United States; 9 The Janssen Pharmaceutical Companies of Johnson & Johnson, Cape Town, South Africa

**Keywords:** clinical trials, Phase 3, viral suppression, resistance, treatment, Africa, adherence, ARV

## Abstract

**Background:**

Evaluating long-term efficacy, safety and pharmacokinetics of long-acting cabotegravir + rilpivirine (CAB+RPV LA) in sub-Saharan African populations is important because of the region’s unique demographics and antiretroviral therapy resistance patterns.

**Objectives:**

To describe the 96-week efficacy, safety and pharmacokinetics of CAB+RPV LA in South African participants from the pooled FLAIR and ATLAS-2M Phase 3/3b randomised studies.

**Method:**

Primary endpoint: proportion of participants with plasma HIV-1 RNA levels ≥ 50 copies/mL at Week 96. Secondary endpoints: proportion of participants with plasma HIV-1 RNA levels < 50 copies/mL, confirmed virological failure (CVF; two consecutive plasma HIV-1 RNA ≥ 200 copies/mL), adverse events and pharmacokinetics.

**Results:**

Sixty-six participants were included, (CAB+RPV LA, *n* = 49; current oral antiretroviral regimen [CAR], *n* = 17). Forty-five (92%) on CAB+RPV LA and 15 (88%) on CAR maintained HIV-1 RNA levels < 50 copies/mL. At Week 96, two participants, one in each arm, had CVF. Ninety per cent on CAB+RPV LA and 76% on CAR of participants experienced an adverse event; six (12%) of which were drug-related (CAB+RPV LA: *n* = 6). Injection-site reactions were common (78% [Grade 1: 80%; Grade 2: 20%]). CAB and RPV trough plasma concentrations remained above respective in vitro protein-adjusted 90% inhibitory concentrations following all doses.

**Conclusion:**

This subgroup analysis of South African participants demonstrated durable efficacy, acceptable safety profile and pharmacokinetics of injectable CAB+RPV LA up to 96 weeks, consistent with long-term data from other regions and studies.

**What this study adds:** This analysis supports the long-term efficacy, safety and pharmacokinetics of CAB+RPV LA in South African participants. Virological suppression, pharmacokinetics, and the safety profile were consistent with those observed in other populations.

## Introduction

Long-acting (LA) injectable antiretroviral therapy (ART), such as LA injectable cabotegravir + rilpivirine (CAB+RPV LA), is an innovative alternative to daily oral ART and is approved as an option for HIV treatment in regions such as Australia, Canada, the European Union, and the United States.^1,2,3,4,5^ Administered intramuscularly (IM) monthly (Q4W) or every 2 months (Q8W), CAB+RPV LA has demonstrated non-inferior efficacy and safety compared to daily oral ART in clinical trials and real-world studies.^6,7,8,9,10,11,12,13,14,15^ Ongoing research suggests benefits for people living with HIV (PLWH) facing adherence barriers or with a history of virologic non-response or loss to follow-up.^16^ While abundant data exist from high-income countries^6,12,13,17^ and early data from sub-Saharan Africa demonstrate efficacy,^14^ longer-term data are needed to assess the durability of this regimen in sub-Saharan Africa including South Africa (SA).

SA has a high HIV burden, with a 17.8% prevalence (predominantly HIV-1 subtype C)^18^ in 2022,^19^ and over five million people receiving ART.^20^ Females are disproportionately affected,^21,22^ representing 63% of new diagnoses in those over 15 years old.^19^ In contrast, HIV incidence in females in high-income regions is lower, ranging from 19% in the United States^23^ to 35.3% in Europe.^24^ Additionally, in SA, the average body mass index (BMI) of females with HIV aged 20–49 years is high, at 27.3 kg/m^2^, which could impact the pharmacokinetics of LA drugs, as has been reported for cabotegravir but not rilpivirine.^25,26,27^

In SA, prior extensive use of non-nucleoside reverse transcriptase inhibitors (NNRTIs) as part of treatment and prevention of vertical transmission ART programmes has contributed to an estimated pre-treatment NNRTI resistance prevalence of 11% in 2016, with a yearly increase estimated at 23%.^28^ Reassuringly, recent 48-week data from the CARES study, which included participants with baseline archived RPV resistance (determined retrospectively),^14^ demonstrated non-inferiority of CAB+RPV LA compared to daily oral ART in sub-Saharan Africa. Notably, 98% of participants achieved HIV-1 RNA < 50 copies/mL in both treatment arms, with only 0.4% in the CAB+RPV LA arm experiencing confirmed virological failure (CVF). Adherence to CAB+RPV LA was high, with 96% of injection visits administered on time.^14^

Given the unique demographic and ART resistance profiles in this population,^29^ additional data on the long-term efficacy and safety of CAB+RPV LA are important.

To describe the long-term efficacy, safety and pharmacokinetics of CAB+RPV LA in SA, we present a post-hoc Week 96 subgroup analysis of the SA cohort enrolled in Phase 3/3b studies, FLAIR (NCT02938520) and ATLAS-2M (NCT03299049), conducted across nine South African study sites.^12,13^

## Research methods and design

### Study design and participants

FLAIR is a randomised, multicentre, parallel-group, open-label, Phase 3 study conducted in 11 countries,^30^ designed to compare the efficacy of CAB+RPV LA Q4W with current antiretroviral oral regimen (CAR; 50 mg dolutegravir, 600 mg abacavir, 300 mg lamivudine) in ART-naïve adults with HIV-1.^7^ Participants were screened between 27 October 2016 and 24 March 2017. They underwent a 20-week induction phase with fixed-dose CAR to achieve virological suppression before being randomised 1:1 to continue the CAR regimen or switch to CAB+RPV LA Q4W (cabotegravir 400 mg and rilpivirine 600 mg as IM injections).^7^

ATLAS-2M is a randomised, multicentre, open-label, Phase 3b study conducted in 13 countries, comparing Q4W versus Q8W administration of CAB+RPV LA in virally suppressed adults with HIV-1.^17,31^ Participants were screened between 27 October 2017 and 31 May 2018. Participants included those transitioning from the ATLAS study after Week 52 and newly recruited, virally suppressed participants receiving an oral standard-of-care ART regimen; standard-of-care in SA was tenofovir disoproxil fumarate, lamivudine, and efavirenz.^17^ Participants were randomised 1:1 to receive either CAB+RPV LA Q8W (CAB 600 mg and RPV 900 mg, 3 mL each) or Q4W (CAB 400 mg and RPV 600 mg, 2 mL each).^17^ RPV was stratified by sex at birth and baseline HIV-1 RNA (< 100 000 or ≥ 100 000 copies/mL) for FLAIR, and previous CAB+RPV (oral plus IM exposure [0, 1–24 or > 24 weeks]) for ATLAS-2M.^7,17^ Participants in the CAB+RPV LA arm of FLAIR and those with no previous exposure to CAB+RPV in ATLAS-2M received an oral lead-in of CAB+RPV for at least 4 weeks.^7,17^

The full methods for these studies have been published previously.^7,17^ Both studies were conducted in accordance with the principles of the Declaration of Helsinki.^7,17^ The main studies were approved by a national, regional ethics committee or institutional review board, as per site and local regulations for each site.^7,17^ All participants signed informed consent prior to being screened and enrolled on the parent studies.^7,17^ As this was a secondary analysis of data collected from main studies, no additional ethical approval was sought.

This analysis pooled 96-week data from FLAIR^12^ and ATLAS-2M (only participants newly recruited were included in this analysis; those transitioning from the ATLAS study to ATLAS-2M were excluded),^13^ focusing on SA participants.

### Outcomes

The primary endpoint of this pooled analysis in SA participants was the proportion of participants with plasma HIV-1 RNA levels ≥ 50 copies/mL at Week 96 in the intention-to-treat exposed population (using the Food and Drug Administration Snapshot algorithm). Secondary endpoints included the proportion of participants with plasma HIV-1 RNA levels < 50 copies/mL, the proportion with CVF (defined as two consecutive plasma HIV-1 RNA ≥ 200 copies/mL), reasons for study withdrawal, treatment preference, visit adherence, treatment-emergent genotypic resistance associated with CVF, safety (including the incidence and severity of adverse events [AEs], clinical laboratory tests, vital signs, electrocardiograms, physical examinations, injection site reactions, and others as previously reported),^7^ and CAB+RPV LA pharmacokinetic profiles (trough plasma concentrations were summarised over time and compared with the protein-adjusted 90% inhibitor concentration).

### Statistical analysis

Statistical methods and population analyses for the FLAIR and ATLAS-2M studies at Week 96 have been published previously.^13,17^ For the current Week 96 subgroup analysis, no significance tests were conducted because of the limited sample size of the SA cohort. Descriptive data are presented for intention-to-treat exposed populations.

### Ethical considerations

The main FLAIR and ATLAS-2M studies were approved by a national, regional ethics committee or institutional review board, as per site and local regulations for each site, and were conducted in accordance with the principles of the Declaration of Helsinki. South African sites were approved by the University of the Witwatersrand: Human Research Ethics Committee (Medical); study approval numbers: 161001 (FLAIR) and 171003 (ATLAS-2M). All participants provided written informed consent prior to being screened and enrolled in the parent studies. All study information was labelled with a code number and did not contain a participant name or address. Only site staff have access to identifiable information. The original studies were conducted in over 120 sites across 13 countries. As this was a secondary analysis of data collected from main studies, no additional ethical approval was required. The clinical trial reference numbers are NCT02938520 (FLAIR) and NCT03299049 (ATLAS-2M).

## Results

Of the 1611 participants randomised in FLAIR (*n* = 566) and ATLAS-2M (*n* = 1045) studies, 66 participants constituted the SA cohort (32 from FLAIR and 34 from ATLAS-2M), with 49 participants (74%) randomised to receive CAB+RPV LA.^12,13^ Twenty-eight participants received Q4W dosing and 21 participants received Q8W dosing. Most participants in the SA cohort CAB+RPV LA arms were female (*n* = 33, 67%) and black people (*n* = 48, 98%); median BMI was 27 kg/m^2^ (Table 1).

**TABLE 1:** Participant baseline characteristics in the South Africa cohort.

Characteristics	ABC/DTG/3TC CAR (*n* = 17)				CAB+RPV LA Q8W IM (*n* = 21)				CAB+RPV LA Q4W IM (*n* = 28)				CAB+RPV LA Total IM (*n* = 49)			
	Median	IQR	*n*	%	Median	IQR	*n*	%	Median	IQR	*n*	%	Median	IQR	*n*	%
Age (years)	32	29–35	-	-	44	32–55	-	-	42	31–49	-	-	43	31–50	-	-
Age ≥ 50 years	-	-	0	0	-	-	6	29	-	-	7	25	-	-	13	27
Female sex at birth	-	-	10	59	-	-	11	52	-	-	22	79	-	-	33	67
**Race**																
White people	-	-	0	0	-	-	0	0	-	-	0	0	-	-	0	0
Black people	-	-	17	100	-	-	20	95	-	-	28	100	-	-	48	98
Asian people	-	-	0	0	-	-	1	5	-	-	0	0	-	-	1	2
People of other races	-	-	0	0	-	-	0	0	-	-	0	0	-	-	0	0
BMI (kg/m^2^)	23	21–26	-	-	25	22–32	-	-	29	23–37	-	-	27	22–35	-	-
Median CD4+ cell count, cells/mm^3^	618	498–700	-	-	604	472–945	-	-	554	456–749	-	-	594	457–795	-	-

3TC, lamivudine; ABC, abacavir; BMI, body mass index; CAB+RPV LA, long-acting cabotegravir and rilpivirine; CAR, current (oral) antiretroviral regimen; DTG, dolutegravir; IM, intramuscularly; IQR, interquartile range; Q4W, every 4 weeks; Q8W, every 8 weeks.

In the intention-to-treat exposed population, 45 (92%) participants in the pooled CAB+RPV LA arms and 15 (88%) in the CAR arm maintained HIV-1 RNA levels < 50 copies/mL at Week 96. Virological data were missing for four participants at Week 96 because of withdrawal from the study (CAB+RPV LA, *n* = 3; CAR, *n* = 1; because of AEs, physician decision, or adherence issues) (Table 2). One participant from the CAB+RPV LA arm (Q8W; 2%) and one participant in the CAR arm (6%) had HIV-1 RNA levels ≥ 50 copies/mL (Table 2).

**TABLE 2:** Study outcomes (Snapshot analysis intention-to-treat exposed) and safety overview in South African participants in FLAIR and ATLAS-2M through 96 weeks.

Outcomes	ABC/DTG/3TC CAR		CAB+RPV LA Q8W IM		CAB+RPV LA Q4W IM		CAB+RPV LA Total IM	
	*n*	%	*n*	%	*n*	%	*n*	%
**Snapshot outcomes (ITT-E)**	**17**	**100**	**21**	**100**	**28**	**100**	**49**	**100**
HIV-1 RNA < 50 copies/mL	15	88	18	86	27	96	45	92
HIV-1 RNA ≥ 50 copies/mL	1	6	1	5	0	0	1	2
CVF†	0	0	1	5	0	0	1	2
No virological data‡	1	6	2	10	1	4	3	6
**Safety overview (excluding ISRs)**	**17**	**100**	**21**	**100**	**28**	**100**	**49**	**100**
Any AE	13	76	18	86	26	93	44	90
Any AEs leading to discontinuation	0	0	1	5	0	0	1	2
Any Grade 3/4/5 AE	0	0	2	10	0	0	2	4
Any drug-related AEs leading to discontinuation	0	0	0	0	0	0	0	0
Any drug-related AE	0	0	1	5	5	18	6	12
Any Grade 3/4/5 drug-related AE	0	0	0	0	0	0	0	0
Any serious AEs	1	6	2	10	-	-	2	4
Drug-related SAEs	0	0	0	0	0	0	0	0
**Treatment preference§**	**-**	**-**	**21**	**100**	**28**	**100**	**49**	**100**
Injectable	-	-	17	81	28	100	45	92
Daily oral	-	-	0	0	0	0	0	0
No preference	-	-	0	0	0	0	0	0
No response	-	-	4	19	0	0	4	8
**Injections**	**-**	**-**	**222**	**100**	**635**	**100**	**857**	**100**
Missed	-	-	0	0	0	0	0	0
**ISRs¶**	**-**	**-**	**298**	**100**	**1134**	**100**	**1432**	**100**
Any ISR	-	-	79	27	82	7	161	11
Any ISR leading to withdrawal	-	-	0	0	0	0	0	0
Grade 1 ISRs	-	-	59	20	69	7	128	9
Grade 2 ISRs	-	-	20	7	13	1	33	2
Grade 3 ISRs	-	-	0	0	0	0	0	0

3TC, lamivudine; ABC, abacavir; AE, adverse event; CAB, cabotegravir; CAR, current (oral) antiretroviral regimen; CVF, confirmed virological failure; DTG, dolutegravir; IM, intramuscularly; ISR, injection site reaction; ITT-E, intention-to-treat exposed; LA, long-acting; NA, not applicable; Q4W, every 4 weeks; Q8W, every 8 weeks; RPV, rilpivirine; SAE, serious adverse event.

† , CVF defined as two consecutive plasma HIV-1 RNA ≥ 200 copies/mL;

‡ , One participant from the CAB+RPV LA Q8W arm withdrew because of an AE, three participants withdrew for other reasons (two from the CAB+RPV LA arms, one from the CAR arm);

§ , Treatment preference data were from 48 weeks, and the survey was not conducted at 96 weeks;

¶ , ISR events/number of injections; ISRs include injection site pain, nodules, swelling, bruising, induration, pruritis, erythema or granuloma.

The single participant in the CAB+RPV LA arm (Q8W) with HIV-1 RNA levels ≥ 50 copies/mL also experienced CVF at Week 16 (despite on-time injections). This participant was a 35-year-old female with a BMI of 44 kg/m^2^ (1.5” needle used) and HIV-1 subtype C, who had previously been treated with efavirenz, emtricitabine, and tenofovir. Genotyping at Week 16 revealed NNRTI resistance-associated mutation (Y188L) and integrase resistance-associated mutations (N155N/H, Q148Q/R), along with an integrase polymorphism (L74L/I). Phenotypic analysis at Week 16 indicated reduced sensitivity to rilpivirine, nevirapine, efavirenz and etravirine, as well as reduced sensitivity to cabotegravir, raltegravir and elvitegravir, with preserved susceptibility to dolutegravir. Retrospective proviral DNA genotyping of baseline stored peripheral blood mononuclear cells revealed a pre-existing integrase resistance-associated mutation (G140G/R), integrase polymorphism L74L/I and major NNRTI resistance-associated mutations (Y188Y/F/H/L). The participant was prescribed LPV/RTV/FTC/TDF and subsequently withdrew from the study.

Five withdrawals occurred in this cohort. Three withdrawals occurred in the CAB+RPV LA Q8W arm: one because of an AE (non-drug-related), one because of CVF, and one because of physician decision (participant had pulmonary tuberculosis); one occurred in the CAB+RPV LA Q4W arm because of participant choice (planned pregnancy); and one occurred in the CAR arm because of physician decision (non-compliant with treatment or protocol procedures).

No injection visits were missed, with 857 (100%) injection visits taking place within the scheduled timeframe of ± 7 days. Regarding treatment preference, of the 45 of 49 participants receiving CAB+RPV LA who responded to a survey, all (100%) favoured CAB+RPV LA over daily oral treatment.

No new safety signals emerged in this SA cohort (Table 2). ISRs were reported 161 times, all of which were Grade 1 (80%, *n* = 128) or Grade 2 (20%, *n* = 33). Injection-site pain was the most common ISR, occurring in 131 of 161 ISR events (81%). Median ISR duration was 3 days, typically resolving within 7 days (83%, *n* = 134). The incidence of ISRs decreased over time, with only four participants (8%) experiencing ISRs at Week 96.

Non-ISR AEs were reported by 90% of participants in the CAB+RPV LA arm and 76% of participants in the CAR arm. Six (12%) drug-related AEs occurred in the CAB+RPV LA arms (mostly dizziness or headaches); none occurred in the CAR arm. Two serious adverse events (SAEs) were reported in the CAB+RPV LA arm, comprising a case of pneumonia and a case of acute hepatitis B. No drug-related SAEs were reported. Commonly reported AEs in the CAB+RPV LA arms included respiratory tract infections (55%) and influenza (14%).

**FIGURE 1:**
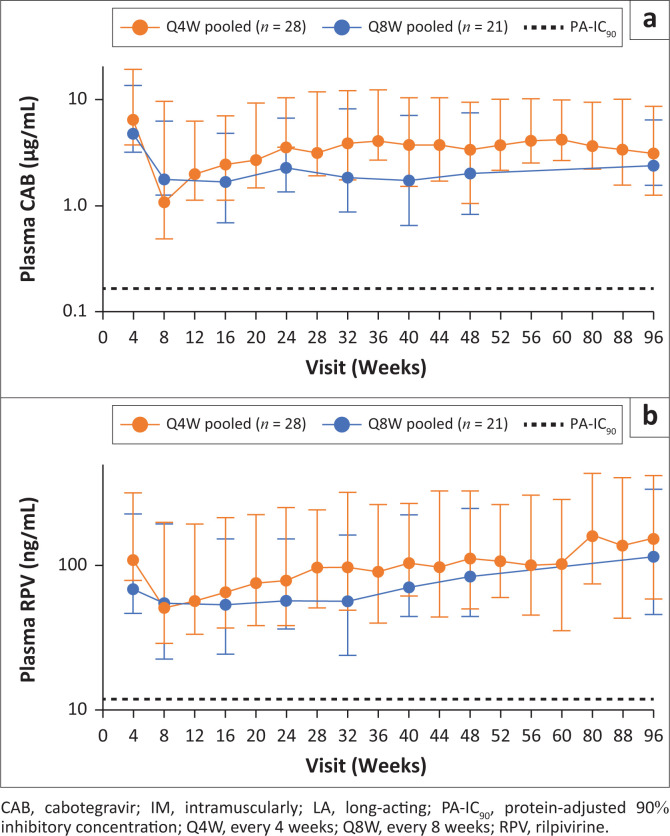
Median (5th and 95th percentile) trough plasma concentration-time (a) CAB and (b) RPV pharmacokinetic profiles following CAB+RPV LA IM Q4W or Q8W: pooled data from South African participants in FLAIR and ATLAS-2M studies.

Median CAB and RPV pharmacokinetic trough concentrations remained above the respective in vitro protein-adjusted 90% inhibitory concentration (PA-IC_90_) values (CAB, 0.166 µg/mL; RPV, 12 ng/mL) at all visits up to Week 96 (Figure 1): at least 10.5-fold (8-week dosing group) and 6.4-fold (4-week dosing group) for CAB, and at least 4.5-fold (8-week dosing group) and 4.2-fold (4-week dosing group) for RPV. At Week 96, the median CAB pre-dose concentrations were 2.4 µg/mL (5th–95th percentile: 0.8 µg/mL – 4.0 µg/mL) for the 8-week dosing group, and 3.2 µg/mL (5th–95th percentile: 1.9 µg/mL – 5.5 µg/mL) for the 4-week dosing group. Median RPV pre-dose concentrations at Week 96 were 115 ng/mL (5th–95th percentile: 69 ng/mL – 220 ng/mL) for the 8-week dosing group, and 152 ng/mL (5th–95th: 93 ng/mL – 265 ng/mL) for the 4-week dosing group.

## Discussion

This analysis of the SA cohort from the FLAIR and ATLAS-2M trials, involving participants switching from daily oral ART regimens to Q4W or Q8W CAB+RPV LA, supports the durable long-term efficacy and an acceptable safety and pharmacokinetic profile of injectable CAB+RPV LA at 96 weeks.

Although the small sample size prevented comparative analyses, these findings align with the overall Week 96 FLAIR and ATLAS-2M analyses, ranging from 87% (Q4W, FLAIR) to 91% (Q8W, ATLAS-2M).^13,17^ In the SA cohort, 92% of participants in the pooled CAB+RPV LA arms maintained virological suppression at Week 96, with only one CVF occurring, despite the lack of prospective baseline genotyping and high historical NNRTI use in SA populations.

These 96-week efficacy, safety and pharmacokinetic data from the SA cohort also support recent findings from the CARES study, which was conducted under a public health approach with an 8-weekly dosing regimen and less frequent (6-monthly) viral load monitoring, and confirmed non-inferior efficacy and safety of CAB+RPV LA versus daily oral ART up to Week 48 in sub-Saharan Africa,^14^ despite the lack of prospective genotype testing at baseline. Similar to the CARES study,^14^ adherence to CAB+RPV LA in the current subgroup was high, as was acceptability with all participants preferring CAB+RPV LA regimen over daily oral ART.

There was a single CVF case in this subgroup analysis. A prior multivariable analysis evaluating predictors of treatment failure based on a broader data set from several CAB+RPV LA registrational trials identified three baseline factors that increased the risk of CVF,^32^ the presence of RPV resistance-associated mutations, HIV-1 subtype A6/A1, and BMI ≥ 30 kg/m^2^. None of these as a single factor predicted virologic failure, but in combination resulted in an increased risk of CVF.^32^ In this SA case of CVF, only one of these baseline predictors was present – BMI ≥ 30 kg/m^2^. In addition, at baseline this participant had a G140R integrase inhibitor (INSTI) resistance mutation, G140R is a nonpolymorphic mutation and can reduce CAB susceptibility up to seven fold. At treatment failure, a further INSTI resistance mutation emerged – Q148H/K/R – which nearly always occurs in combination with G140A/S or E138K and confers high-level resistance to raltegravir, elvitegravir, and CAB, and low-to-intermediate reductions in dolutegravir and bictegravir susceptibility.^33,34^ Further evidence on optimal ART sequencing for those who develop CVF may help inform the future use of CAB+RPV LA in settings where INSTI-based ART regimens are the cornerstone of national HIV treatment programmes.

In cases where participants have a high BMI and thicker layer of adipose tissue, 2-inch needles have been recommended for administration of CAB+RPV LA; it is noteworthy that in this case of CVF a shorter needle length (1.5 inches) was used to administer the regimen.

Although the SA cohort predominantly consisted of black (98%) female participants (67%) with a high BMI (median 27 kg/m^2^), the safety and pharmacokinetic profiles appeared to align with wider trial populations which primarily comprised white male participants (> 70%) with lower BMIs (24 kg/m^2^ – 25.9 kg/m^2^).^7,17^ Pharmacokinetic profiles of CAB and RPV remained above their respective PA-IC_90_ values at all visits through Week 96. Though limited by the small sample size in the SA cohort, trough concentrations were largely in line with those previously reported for the overall study populations. Week 96 geometric mean trough concentrations for the 4-week dosing regimen in FLAIR were 2.8 µg/mL for CAB and 112 ng/mL for RPV.^12^ In ATLAS-2M (participants with no prior exposure from ATLAS), Week 96 geometric mean trough concentrations were 1.57 µg/mL for cabotegravir and 86 ng/mL for rilpivirine for the 8-week regimen, and 2.75 µg/mL for CAB and 121 ng/mL for RPV for the 4-week regimen.^13^ Race has, to date, not been identified as a significant covariate in the population pharmacokinetic analysis for either compound.^26,27^

Similar to prior trials, ISRs were common, but mostly of mild or moderate severity (Grade 1 or Grade 2), with decreasing incidence over time. In ATLAS-2M at 96 weeks there were 6% SAEs in the 8-week dosing arm and 5% in the 4-week dosing arm, which led to discontinuation in < 1% (Q8W) and 1% (Q4W) of participants.^13^ In the SA subgroup, similarly, at 96 weeks there were 4% SAEs in the pooled LA arms, with none leading to discontinuation. The single case of acute Hepatitis B highlights the need to consider how ART regimens that do not have anti-Hepatitis B activity may be introduced in regions of high Hepatitis B endemicity.

Our findings provide some insights into the long-term pharmacokinetics, efficacy and safety of CAB+RPV LA in SA participants, and aligns with data from the CARES study, which reported non-inferior efficacy of CAB+RPV LA against oral ART in sub-Saharan Africa populations.^14^

The limitations of the FLAIR and ATLAS-2M studies have been reported previously and included lack of blinding, potentially resulting in patients anticipating and reporting more AEs because of the administration of CAB+RPV LA, and possibly safety assessments performed more frequently for the Q4W arm compared with the Q8W arm, because of the difference in dosing frequency. Furthermore, this pooled analysis was limited to a small sample size, and thus no statistical tests were performed. The efficacy and safety findings of this study are therefore descriptive in nature.

## Conclusion

This descriptive SA subgroup analysis of Phase 3/3b CAB+RPV LA clinical trials supports the durable efficacy, and acceptable safety and pharmacokinetic profiles in virologically suppressed SA participants up to Week 96, consistent with the reported findings in the overall population and emerging data on the use of this regimen in sub-Saharan African populations. Future data on efficacy in a wider range of participants, including those with challenges with adherence, will provide further insights.^35^
